# Thermogravimetric Analysis of Modified Montmorillonite Clay for Mycotoxin Decontamination in Cereal Grains

**DOI:** 10.1155/2020/6943514

**Published:** 2020-11-28

**Authors:** Bunmi K. Olopade, Obinna C. Nwinyi, Joseph A. Adekoya, Isiaka A. Lawal, Olushola A. Abiodun, Solomon U. Oranusi, Patrick B. Njobeh

**Affiliations:** ^1^Department of Biological Sciences, College of Science and Technology, Covenant University, Km 10, Idiroko Road, Ota, Ogun, Nigeria; ^2^Department of Biotechnology and Food Technology, University of Johannesburg, Doornfontein Campus, Johannesburg, Gauteng 2028, South Africa; ^3^Department of Chemistry, College of Science and Technology, Covenant University, Km 10, Idiroko Road, Ota, Ogun, Nigeria; ^4^Vaal University of Technology, Vanderbijlpark Campus, Boulevard, Vanderbijlpark 1900, South Africa; ^5^Department of Biological Oceanography, Nigerian Institute for Oceanography and Marine Research, Victoria Island, Lagos 101241, Nigeria

## Abstract

Thermogravimetric analysis (TGA) was carried out to study the stability of nanoformulations used for the decontamination of mycotoxins. The TGA patterns of the nanoformulations from montmorillonite clay and *Cymbopogon citratus* (lemongrass) extracts were assessed with temperature ranging from ambient (20°C) to 1000°C. The various nanoformulations studied included unmodified montmorillonite clay (Mont), montmorillonite washed with sodium chloride (Mont-Na), montmorillonite mixed with lemongrass essential oil (Mont-LGEO), and montmorillonite mixed with an equal quantity of lemongrass powder (Mont-LGP). There was no significant difference in the median of the various nanoformulations within 4 weeks at *p* < 0.05 using the Kruskal–Wallis nonparametric test. For the TGA, the first degradation for montmorillonite clay and the nanoformulations occurred at a temperature between 80 and 101°C and was attributed to the loss of lattice water outside the coordination sphere with a range of 3.5–6.5% weight loss. The second degradation occurred within the temperature of 338 to 344°C, and the third, at a temperature between 640 and 668°C for Mont and the formulations of Mont-Na, Mont-LGEO, and Mont-LGP. There were strong similarities in the degradation patterns of Mont and Mont-Na with the minimum difference being the relatively higher weight loss of the sodium-exchanged cation for Mont-Na at the third degradation step. Hence, the order of stability from the most resistant to the least resistant to degradation is as follows: Mont-LGEO ≥ Mont-Na ≥ Mont ≥ Mont-LGP.

## 1. Introduction

Montmorillonite clay has been used for several applications, including the adsorption of mycotoxins in feeds [1]. The cytotoxic properties of montmorillonite clay are insignificant, making it readily efficient for use as excipients in oral and topical drugs [2]. The space between the layers of montmorillonite makes up about 90% of its entire surface. Hence, it enhances the ability of clay to absorb molecules of water and other polar molecules [3, 4].

The main mycotoxins of public concern that often occur in cereal grains such as maize are aflatoxins, deoxynivalenol, fumonisins, ochratoxins, and zearalenone [5, 6]. High incidences of mycotoxins, such as fumonisins and zearalenone in maize, have also been reported [7, 8].

Different types of clay, including bentonite, have been employed for the decontamination of mycotoxins in food and feed [9], thus serving as mycotoxin binders. Other commercial mycotoxin binders employed in feeds include Mycosorb, Formycin, and Anzymit, amongst others [10]. *Cymbopogon citratus* (lemongrass) powder and extracts are equally effective in decontaminating mycotoxins in crops [11–13]. Furthermore, a mixture of montmorillonite clay and lemongrass has been applied for the same purpose in cereal grains. Modified montmorillonite clay has proven efficient in decontaminating T-2 toxin in maize and zearalenone in millet [14, 15]. The use of clay for decontamination of mycotoxins is an adsorption approach, which involves chemical and physical forces that help decrease the formation of toxins and lessen their health effects thereof in animals [16]. In a bid to validate the use of nanoformulations from clay for mycotoxin decontamination, it is essential to ensure that such nanoformulations remain viable and not depleted. This study, therefore, aimed to investigate the stability of various nanoformulations made from montmorillonite clay combined with powder and extracts of lemongrass.

## 2. Materials and Methods

### 2.1. Sample Preparation

Montmorillonite K10 powder (CAS number 1318-93-0) purchased from Sigma-Aldrich, Germany, was used, while the leaves of *Cymbopogon citratus* utilised in the study were identified at the Botany Unit of the Department of Biological Sciences, Covenant University, Nigeria. Lemongrass (*Cymbopogon citratus*) leaves were cleaned using distilled water and air-dried at 25°C for three weeks. After drying, the leaves were pulverised using an electric blender (IKA M20, Wilmington, NC, USA). Soxhlet extraction method, as described by Ojewumi et al. [17], was used to obtain the extracts from lemongrass. For extraction, 250 mL of hexane was added to 25 g of the lemongrass powder and extracted using a rotary evaporator (IKARV10, Staufen, Germany). The concentrated crude essential oil was used to prepare Mont-LGEO according to the modified method of Noudem et al. [18]. The nanoformulations used for the decontamination of AFG_1_ in millet were prepared as described by Olopade et al. [14]. The decontamination process for these two toxins was studied by mixing the nanoformulations with the cereal grains and storing over four weeks. The same approach was applied for the decontamination of AFG_1_ in millet in this study. The preparation of Mont-LGEO involved the homogenisation of montmorillonite clay with sodium chloride solution (1 mol L^−1^) (1 : 20, w/v) at 25°C followed by dialysation of the mixture in deionized water to get rid of chloride ions. The mixture was centrifuged at 50 rpm for 5 min, and the supernatant decanted, while the resulting dialysed mixture was dried at 40°C in an oven to allow water evaporation from the clay. Montmorillonite was mixed with 50 mL of essential oil solution (v/v) prepared using acetone. The essential oil solution was prepared by dissolving 5 mL of the crude essential oil in 45 mL of acetone. The mixture was homogenised at 25°C in an overhead shaker at 50 rpm and then secured in a hot water bath set at 60°C for 90 min to dry out acetone. The mixture (Mont-LGEO) was dried in an oven overnight at 30°C and then stored in tightly sealed coloured vials. For the Mont-Na mixture, montmorillonite clay was modified by the homogenisation of montmorillonite clay with NaCl solution (1 mol L^−1^) (1 : 20, w/v). The last formulation, Mont-LGP, was prepared by mixing montmorillonite with an equal quantity of lemongrass powder (Mont-LGP).

### 2.2. Thermogravimetric Analysis of Modified Clay

Thermogravimetric analysis of the various types of modified clay was carried out with the aid of STA7200RV Thermal Analysis system. Each powder sample was placed in an alumina crucible (6 mm diameter). A constant heating rate of 5°C/min was applied from 20 to 1000°C under nitrogen atmosphere with a flow rate of 50 mL/min.

### 2.3. Quantification of AFG_1_ before Treatment

To determine the apparent recovery (AR) for AFG_1_, concentrations of AFG_1_ (0.25 and 1 *µ*L of the AFG_1_ reference standard) were spiked to a blank within a calibration range of 156.25–5000 *µ*g/kg. The limit of detection (LOD) and limit of quantification (LOQ) were also determined. The mycotoxin (AFG_1_) in millet was analysed by liquid chromatography-tandem mass spectrometry (LC-MS/MS), described by Sulyok et al. [19]. The extraction of AFG_1_ was performed in a 50 mL polypropylene tube (Sarstedt, Nümbrecht, Germany) by homogenising 5 g of the pulverised millet sample with an extraction solvent (20 mL) containing acetonitrile/water/formic acid (79 : 20 : 1, v/v/v) for 90 min on a GFL 3017 rotary shaker (GFL, Burgwedel, Germany). The filtrate (500 *µ*L) was then injected into the LC-MS/MS system to determine the level of AFG_1_.

### 2.4. Decontamination of AFG_1_ in Millet

The millet samples were treated with the nanoformulations (Mont, Mont-Na, Mont-LGEO, and Mont-LGP) at 8% and 12% concentrations in duplicates and put in storage for 4 weeks at a temperature of 30°C in the incubator according to the modified method of Atanda and Olopade [12]. The control (untreated millet sample) from the initial batch was also stored at 30°C just as the treated millet samples. Quantification of the levels of AFG_1_ in the treated millet samples was carried out weekly for up to four weeks according to the LC-MS/MS method of Sulyok et al. [19].

### 2.5. Data Analysis

IBM SPSS statistics software (ver. 23, 2015 Inc., Chicago, IL, USA) was used to perform a normality test on the results obtained, after which the nonparametric test was performed to compare the median of the various nanoformulations analysed in duplicates within 4 weeks to check for significance at *p* < 0.05 since the data was not a normal distribution. Thermogravimetric analysis curves were generated by using the Origin software (version 8.5, OriginLab Corporation Northampton, MA, USA) to plot the weight loss in the nanoformulations against the temperature of the nanoformulations when heated.

## 3. Results and Discussion

### 3.1. Thermogravimetric Analysis (TGA) of the Nanoformulations from Montmorillonite Clay and Lemongrass Extracts

**Figure 1:**
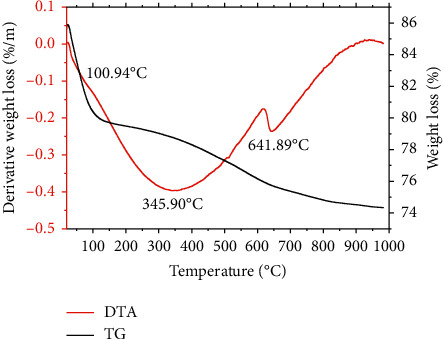
TGA/DTA curves showing the degradation pattern of montmorillonite clay.

**Figure 2:**
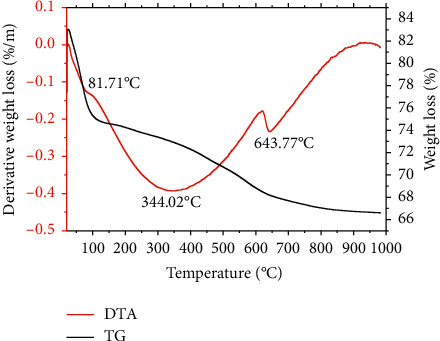
TGA/DTA curves showing the degradation pattern of montmorillonite-Na.

**Figure 3:**
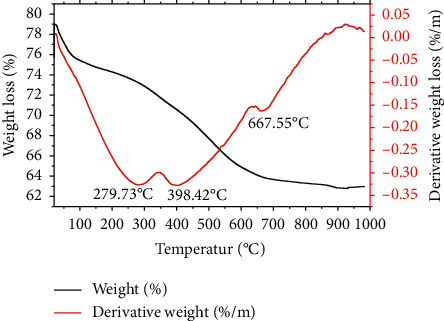
TGA/DTA curves showing the degradation pattern of montmorillonite-lemongrass essential oil.

**Figure 4:**
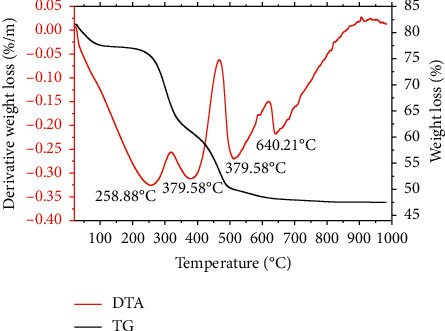
TGA/DTA curves showing the degradation pattern of montmorillonite-lemongrass powder.

Thermogravimetric analysis curves of montmorillonite powder, montmorillonite-Na, montmorillonite-lemongrass essential oil, and montmorillonite-lemongrass powder are shown in Figures 1–4, respectively. The thermogravimetric analysis of the various nanoformulations in this study is very crucial to determine how stable and potent the nanoformulations used for the decontamination of mycotoxins will be over time. The first degradation for montmorillonite K10 was observed at 100.94°C. The second degradation for Mont. was observed at 345.90°C, while the third degradation occurred at 641.89°C (Figure 1). For Mont-Na, the first degradation occurred at 81.71°C, while the second one occurred at 344.02°C, and the third degradation at 643.77°C, as shown in Figure 2. The montmorillonite clay contains water due to lattice water outside the coordination sphere of the spinel clay layers [20]. Therefore, the first degradation for the nanoformulations Mont and Mont-Na occurred at about the same temperature, which was as a result of a loss in the water of hydration in the formulations. The percentage loss in the first and second degradation for Mont. was 6 and 5%, respectively. For Mont-Na, 8.5% weight loss was recorded in the first degradation, while 6.5% weight loss was recorded in the second degradation. There were similarities in the degradation patterns of Mont. and Mont-Na. The visible change was the loss of water of hydration which occurred from 100°C in unmodified montmorillonite clay to 87.17°C in Mont-Na.

The first, second, and third degradations for Mont-LGEO occurred at 279.73, 398.42, and 667.55°C, respectively with a weight loss of 3.5, 10.0, and 2.5% (Figure 3). For Mont-LGP, the first degradation occurred at 258.88°C, whereas the second degradation occurred twice at 379.58°C and the third at 640.21°C (Figure 4). The weight loss in the first, second, and third degradation for Mont-LGP was 4.5, 18.5, and 8%, respectively. The initial loss of water of hydration in unmodified montmorillonite clay occurred at 100°C. However, the presence of lemongrass in the compositions slowed down the loss of water of hydration within the nanoformulations. Also, montmorillonite comprises tetrahedral silica and octahedral alumina layers, which possess negative charges that can be balanced by exchangeable cations within its interlayers [21].

**Figure 5:**
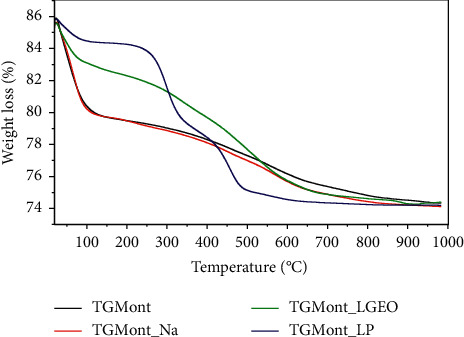
Combined TGA curves showing the degradation patterns of Mont, Mont-Na, Mont-LGEO, and Mont-LGP.

The combined thermogravimetric (TGA) curve in Figure 5 confirmed that the degradation of Mont. and Mont-Na were very similar and occurred at a steady rate. The curve for Mont-LGEO showed that degradation occurred at a slow pace compared with Mont-LGP. The TGA curve for Mont-LGEO showed a gradual degradation, whereas the TGA curve for Mont-LGP showed a fast and sharp irregular degradation pattern. The results indicate that the presence of the admixtures of lemongrass essential oil increases the thermal stability of the nanoformulation. The silicate layers distributed evenly in the montmorillonite sheet exhibited intermolecular interaction with the radical products from the nanoformulations of lemongrass essential oil, which consists mostly of geranial derivatives. Hence, Mont-LGEO will be more stable and potent compared with Mont-LGP. Generally, mycotoxin removal from foods by montmorillonite clay occurs through the mechanism of adsorption [16, 22]. The adsorption process was improved by the exchange of cations between the lemongrass extract and the montmorillonite clay. However, adsorption is a reversible process under unstable conditions [23, 24]. Hence, the TGA of the nanoformulations was performed to determine the most stable nanoformulation that will not be prone to degradation, thereby compromising the mycotoxin decontamination process.

**Figure 6:**
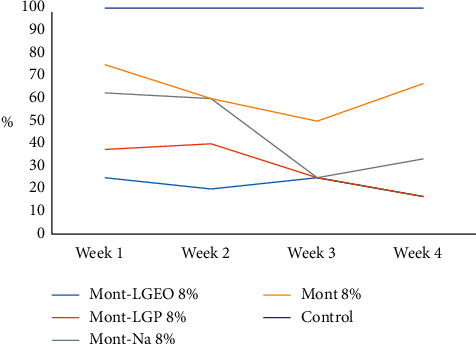
Percentage reduction of AFG_1_ after 8% of each treatment for 4 weeks.

**Figure 7:**
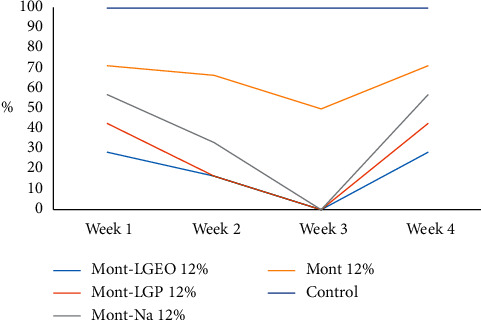
Percentage reduction of AFG_1_ after 12% of each treatment for 4 weeks.

The apparent recovery for AFG_1_ was 100%, while its limit of detection (LOD) and limit of quantification (LOQ) were 0.002 and 0.006 *µ*g/kg, respectively. Figures 6 and 7 show the level of decontamination of AFG_1_ in the millet sample after treatment with 8% and 12% of the nanoformulations for 4 weeks. More decontamination of AFG_1_ was observed after the third week. However, the normality test carried out showed that the data were not normally distributed. Therefore, the Kruskal–Wallis nonparametric test was applied. The nonparametric test showed that there was no significant difference in the median of the various nanoformulations within the first to fourth week of treatment at *p* < 0.05. The control measuring 0.001 *µ*g/kg was constant throughout the four weeks. The nanoformulations did not show any significant difference in the decontamination of AFG_1_ in the millet samples compared with the control. However, in the previous studies, Mont-Na was proven to be the most efficient in decontaminating T-2 toxin in maize [14], while Mont-LGP was the most efficient in decontaminating zearalenone in millet [15]. Other studies have also shown that bentonite which is mainly montmorillonite is effective in the adsorption of mycotoxins, particularly aflatoxins [25, 26].

## 4. Conclusion

This study showed that nanoformulations of montmorillonite clay with *C. citratus* are quite relatively resistant to degradation with the combination of montmorillonite and lemongrass essential oil more resistant than sodium-exchanged montmorillonite and montmorillonite modified with lemongrass powder. Therefore, nanoformulations from montmorillonite clay and lemongrass essential oil can serve as stable mycotoxin-decontaminating agents in stored cereal grains.

## Data Availability

The data used to support the findings of this study are available from the corresponding author upon request.
